# Tuberculosis and lactic acidosis as causes of death in adult patients from a regional hospital in Johannesburg

**DOI:** 10.4102/phcfm.v4i1.266

**Published:** 2012-02-17

**Authors:** Malangu Ntambwe, Mogashoa Maryet

**Affiliations:** 1 Department of Epidemiology, University of Limpopo (Medunsa Campus), South Africa; 2 Center for Diseases Control, South Africa

## Abstract

**Background:**

Tuberculosis and adverse effects have been shown to affect both the quality of life and the survival of patients on antiretroviral treatment. This study sought to investigate the causes of death in a sample of adult HIV-infected patients on antiretroviral treatment at Thembisa Hospital, Johannesburg, South Africa.

**Methods:**

A retrospective study was conducted by examining the charts of 498 adult patients treated from January 2004 to December 2006 at the antiretroviral clinic of a regional hospital in Johannesburg. A data collection form was used to collate both sociodemographic and clinical data.

**Results:**

The majority of the patients were female (71.7%) with a mean age of 37.7 ± 11.6 years, and in the age group of 18–77 years. The greater number of the patients was South African citizens, with only 2.2% citizens of other Southern African countries. At baseline, 29.9% had been on anti-tuberculosis treatment. Most of the patients had been prescribed the regimen comprising stavudine, lamivudine, and nevirapine or efavirenz; two of them (0.4%) were on the second line regimen made of zidovudine, didanosine, and lopinavir–ritonavir. At least one side effect was documented in 82.1% of patients; the ten most documented side effects were skin rashes (62.9%), peripheral neuropathy (48.4%), headaches (38.2%), chest pain (21.9%), coughing (21.7%), anaemia (21.5%), diarrhoea (19.3%), vomiting (16.7%), dizziness (15.3%), and lactic acidosis (11.2%). A mortality rate of 3.6% was recorded during the 2-year study period. Although the cause of death was undetermined in 11.1% of patients, 50.0% and 38.9% of deaths respectively were a consequence of tuberculosis and lactic acidosis.

**Conclusions:**

In addition to tuberculosis, side effects in particular, lactic acidosis was the other main cause of death in patients treated at the study site. These findings suggest that patients on regimens containing drugs that cause lactic acidosis should be closely monitored when the first complaints suggesting lactic acidosis are reported or noticed.

## Abstrait

**La tuberculose et l'acidose lactique comme causes de décès de patients adultes d'un hôpital régional à Johannesburg**

**Contexte:**

Il a été prouvé que la tuberculose et ses effets négatifs affectaient à la fois la qualité de vie et la survie des patients sous traitement antirétroviral. Cette étude a cherché à enquêter sur les causes de décès d'un échantillon de patients adultes séropositifs sous traitement antirétroviral à l'hôpital de Thembisa, à Johannesburg, en Afrique du Sud.

**Méthodes:**

Une étude rétrospective a été réalisée en examinant les carnets de santé de 498 patients adultes traités de janvier 2004 à décembre 2006 dans un centre de traitement antirétroviral d'un hôpital régional à Johannesburg. Un formulaire de collecte des données a été utilisé afin de recueillir des données sociodémographiques et cliniques.

**Résultats:**

La majorité des patients étaient des femmes (71.7%), dont la moyenne d’âge était de 37.7 ± 11.6 ans et qui appartenaient à la tranche d’âge des 18–77 ans. La majorité des patients étaient citoyens sud-africains, 2.2% seulement étant des ressortissants d'autres pays d'Afrique australe. Au début de l’étude, 29.9% avaient suivi un traitement contre la tuberculose. La plupart des patients s’étaient vus prescrire le traitement à base de stavudine, lamivudine, et névirapine ou d’éfavirenz; deux d'entre eux suivaient le traitement de seconde ligne à base de zidovudine, didanosine, et lopinavir–ritonavir. Un effet secondaire au moins a été documenté chez 82.1% des patients; les dix effets secondaires les plus documentés étaient les éruptions cutanées (62.9%), la neuropathie périphérique (48.4%), les maux de tête (38.2%), les douleurs thoraciques (21.9%), la toux (21.7%), l'anémie (21.5%), la diarrhée (19.3%), les vomissements (16.7%), les vertiges (15.3%) et l'acidose lactique (11.2%). Un taux de mortalité de 3.6% a été enregistré au cours de la période d’étude de deux ans. Bien que la cause du décès soit restée indéterminé pour 11.1% des patients, 50.0% et 38.9% des décès étaient respectivement dus à la tuberculose et à l'acidose lactique.

**Conclusion:**

Outre la tuberculose, les effets secondaires, en particulier l'acidose lactique, étaient l'autre cause principale de décès chez les patients traités sur le site de l’étude. Ces conclusions indiquent que les patients suivant un traitement contenant des médicaments qui provoquent une acidose lactique doivent être suivis de près lorsque les premiers signes suggérant une acidose lactique sont signalés ou observés.

## Introduction

The roll-out of antiretroviral treatment in South Africa has led to positive outcomes that have been documented in several studies.^1–3^ Significant reductions in AIDS-related morbidity and mortality have been documented already.^4–6^ Unfortunately, up to 25% of patients discontinue their initial antiretroviral treatment regimen because of treatment failure, non-compliance or low adherence, as well as to adverse effects.^7–10^ Adverse effects have been shown to affect both the quality of life and the survival of patients, and it follows that it is important to describe these effects. In doing so, the findings of this study will contribute to the identification of adverse or side effects that should be targeted for monitoring by clinicians and for creating awareness of these effects amongst patients. The purpose of this study, therefore, was to investigate the causes of death in a sample of adult HIV-infected patients on antiretroviral treatment at Thembisa Hospital in Johannesburg, South Africa.

### Significance of the study

This study investigated the side effects associated with mortality in a sample of adult HIV-infected patients on antiretroviral treatment. The findings of this study will contribute to the identification of side effects that should be targeted for monitoring by clinicians and for creating awareness of these effects amongst patients.

## Ethical considerations

The approval to conduct this study was obtained from the Medunsa Campus Research and the Ethics Committee of the University of Limpopo. Permission to access the patients’ records was requested and obtained from Management at the hospital.

## Methods

A retrospective study was conducted by examining the charts of 498 adult patients treated from January 2004 to December 2006 at the antiretroviral clinic of a regional hospital in Johannesburg. A data collection form was used to collate both sociodemographic and clinical data, including the age and sex of the patients as well as the documented side effects, whether they had died or were still alive, the recorded cause of death, the stage of the infection, the duration of treatment, and the regimen they took. Descriptive statistics and cross-tabulation were used in the analysis of data. All statistical analyses were performed using SPSS software (version 17.0; SPSS, Chicago, IL, USA).

## Results

The sample comprised mostly female patients (71.7%), whose mean age was 37.7 ± 11.6, ranging from 18 to 77 years. The greater number of the patients was South African citizens, with only 2.2% citizens of other Southern African countries such as the Democratic Republic of Congo (1), Malawi (1), Mozambique (1), Zambia (1), and Zimbabwe (7). The majority of patients had completed a high school level of education (75.1%) and were unemployed (80.3%). With regard to their lifestyle, few of them smoked (6.7%), or consumed alcoholic drinks (8%). From the onset of enrolment into the antiretroviral treatment, 29.9% had been on anti-tuberculosis treatment.

**FIGURE 1:**
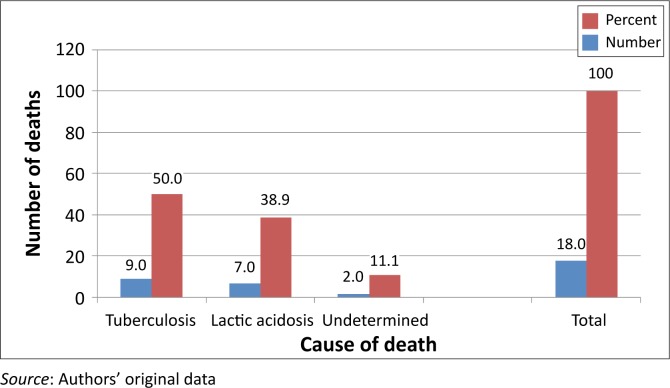
Number and causes of death of HIV-infected patients at Thembisa Hospital 2004–2006.

**FIGURE 2:**
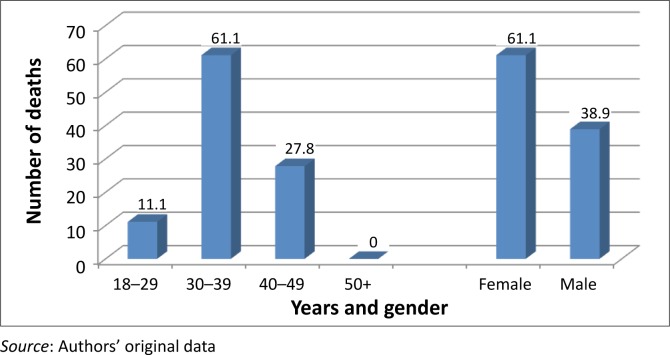
Demographic characteristics of HIV-infected patients that died at Thembisa Hospital 2004–2006.

**FIGURE 3:**
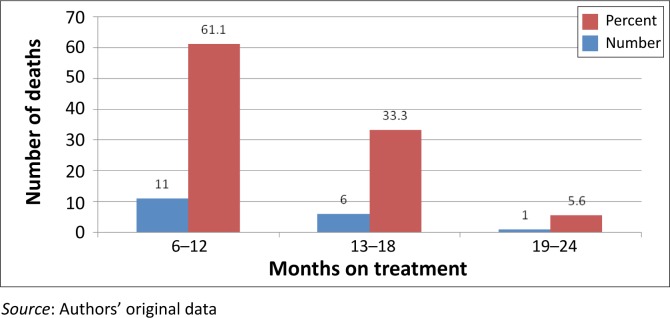
Trends of death per treatment period at Thembisa Hospital 2004–2006.

**FIGURE 4:**
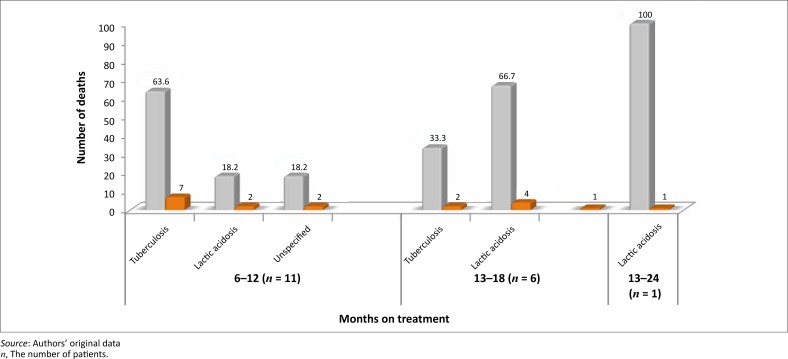
Tuberculosis and lactic acidosis deaths per treatment period at Thembisa Hospital 2004–2006.

Their immunological status was considered as poor because most of them (97.2%) had CD4 counts < 200 copies/mL. The majority (57%) of them were afflicted by severe immune depression because they met the WHO (World Health Organisation) clinical Stage 3 and Stage 4. Most of the patients had been prescribed the regimen made of stavudine, lamivudine, and nevirapine or efavirenz; two of them (0.4%) were on the second line regimen made of zidovudine, didanosine, and lopinavir–ritonavir (Table 1). At least one side effect was documented in 82.1% of patients. The ten most documented side effects (Table 2) were skin rashes (62.9%), peripheral neuropathy (48.4%), headaches (38.2%), chest pain (21.9%), coughing (21.7%), anaemia (21.5%), diarrhoea (19.3%), vomiting (16.7%), dizziness (15.3%), and lactic acidosis (11.2%). Of these side effects, lactic acidosis was associated with a number of deaths. Overall, a mortality rate of 3.6%, or 18 deaths during the 2-year study period, was recorded. Although the cause of death was undetermined in 11.1% of patients, 50.0% and 38.9% of deaths were ascribed in the patients’ records, respectively to tuberculosis and lactic acidosis (Figure 1). The odd ratios of dying from lactic acidosis was 5.6 [(95% CI: 2.1–15.1); *p* = 0.0001]; compared to 4 [(95% CI: 1.5–10.4); *p* = 0.002] for tuberculosis. However, all the 18 patients that died were amongst the 284 that had severe immune suppression. Moreover, 11 of the 18 deaths had a CD4 cells count ≤ 100 copies/mL (Table 3). Although most of the deaths occurred between the first 6 months to the end of the first year of treatment (Figure 3), deaths associated with lactic acidosis continued to occur even after 18 months on treatment (Figure 4). Of those who died, the majority had been initiated on antiretroviral treatment late (they were classified as WHO Stage 3), they were mostly female, and aged 30–39 (Figure 2).

**TABLE 1:** Sociodemographic and baseline data of HIV-infected patients from Thembisa Hospital 2004–2006.

Variables	Frequency	%
**Gender**		
Female	*354*	71.1
Male	144	28.9
**Age group** (years)		
18–29	70	14.1
30–39	244	49.0
40–49	134	26.9
50 +	50	10.0
**Level of education**		
Grades 0–7	108	21.7
Grades 8–12	374	75.1
Tertiary level	16	3.2
**Employment status**		
Unemployed	400	80.3
Employed	98	19.7
**Lifestyle**		
Alcohol drinking status	40	8.0
Smoking status	34	6.8
**Type of regimen**		
Regimen 1a	312	62.7
Regimen 1b	184	36.9
Second line	2	0.4
**Tuberculosis (TB) treatment status**		
On TB treatment	105	29.9
Not on TB treatment	246	70.1
**WHO clinical stage**		
Stage 1	45	9.0
Stage 2	169	33.9
Stage 3	239	48.0
Stage 4	45	9.0
**Viral load (VL) status at baseline**		
VL ≤ 400	8	1.6
VL > 400	478	98.4
**CD4 count status at baseline**		
CD4 > 200	14	2.8
CD4 ≤ 200	484	97.2

*Source*: Authors’ original data

WHO, World Health Organisation.

**TABLE 2:** Side effects documented in HIV-infected patients at Thembisa Hospital 2004–2006.

Side effects	Frequency	%
Skin rashes, itching	313	62.9
Peripheral neuropathy	241	48.4
Headache	190	38.2
Chest pains	109	21.9
Coughing	108	21.7
Anaemia	107	21.5
Diarrhoea	96	19.3
Nausea or vomiting	83	16.7
Dizziness	76	15.3
Lactic acid acidosis	56	11.2
Heartburn	44	8.8
Fatigue	42	8.4
Loss of appetite	40	8.0
Bloating	38	7.6
Weight loss	37	7.4
Muscle and joint pains	33	6.6
Trouble sleeping	25	5.0
Ringing in ear	20	4.0
Blurred vision	20	4.0
Cotrimoxazole allergy	18	3.6

*Source*: Authors’ original data

**TABLE 3:** Clinical stage of HIV-infected patients that died at Thembisa Hospital 2004–2006.

Period	Cause	Variables	WHO stage		Baseline CD4 counts values	
	
			Stage 3	Stage 4	CD4 < 100	CD4 100–200
6th–12th month	Unknown	Patient 1	Yes	-	6	-
		Patient 2	Yes	-	-	100
	Lactic acidosis	Patient 1	-	Yes	31	-
		Patient 2	Yes	-	-	131
	Tuberculosis	Patient 1	Yes	-	-	193
		Patient 2	Yes	-	-	196
		Patient 3	-	Yes	1	-
		Patient 4	-	Yes	9	-
		Patient 5	Yes	-	38	-
		Patient 6	Yes	-	-	169
		Patient 7	Yes	-	71	-
12th–18th month	Lactic acidosis	Patient 1	Yes	-	3	-
		Patient 2	Yes	-	16	-
		Patient 3	Yes	-	-	130
		Patient 4	Yes	-	66	-
	Tuberculosis	Patient 1	-	Yes	85	-
		Patient 2	Yes	-	84	-
18th–24th month	Lactic acidosis	Patient 1	-	Yes	31	-

*Source*: Authors’ original data

## Discussion

The prevalence of adverse events documented was 82.1%. This figure is comparable to the figure of 89% reported at another hospital from the same province.^11^ This finding suggests that health-care providers of antiretroviral therapy have been doing good work by recording the adverse effects noticed. It further corroborates the view that side effects are common during antiretroviral therapy in South Africa in accordance to reports elsewhere.^12–14^ The most common effects, skin rashes, peripheral neuropathy, and lactic acidosis have been consistently reported in patients taking the regimen containing nevirapine and/or stavudine. At the time of the study, almost all patients were prescribed the regimen that contained stavudine. The range of adverse effects documented was similar to that reported previously by other investigators.^11–14^ Moreover, the impact of adverse effects on adherence to antiretroviral treatment is well established;^15, 16^ in this study, their impact on mortality has been assessed.

With regard to mortality, 3.6% had died during the 2 years covered by the study. This finding is consistent but lower than the figures reported in the literature.^17–20^ In fact, it has been reported that 8% – 26% of patients die within the first year of antiretroviral treatment.^21^ Furthermore, the findings of this study concur with previous studies in that most deaths occurred during the initial months of treatment.^22, 23^ Data from this study show that the majority of patients sought treatment when they were at an advanced clinical stage of the disease because 57% of them had been classified as WHO Stage 3 and Stage 4 patients (Table 3). This finding is consistent with previous reports about the influence of late initiation of antiretroviral treatement.^1, 3, 4, 17, 23^ In addition, 29.9% of them were afflicted by tuberculosis. Overall, 50% and 38.8% of deaths reported in this study were ascribed to tuberculosis and lactic acidosis respectively. Although the odds of dying were high for both conditions, because all patients that died had been severely immune-compromised, it is plausible that this situation also contributed to the deaths. Furthermore, although deaths ascribed to tuberculosis decreased from a majority (63.6%) status by the end of the first year of treatment to zero towards the end of the second year, deaths ascribed to lactic acidosis increased during the same periods from 18.2% to 66.7% (Figure 3). This finding suggests that, although deaths caused by tuberculosis could be halted when tuberculosis is cured, the danger from lactic acidosis cannot be averted easily. Stavudine is known for its association with lactic acidosis, and consequently this finding lends support to the decision to remove stavudine as a component of the first line regimen in South Africa.^7, 13, 25, 26^ Indeed, as from April 2010, the new guidelines have replaced stavudine with tenofovir in the first line regimen.^27^ The rationale for this proposition is that lactic acidosis is insidious and produces ordinary symptoms such as nausea, vomiting, and abdominal pain. These symptoms may be overlooked by clinicians, which make it difficult for them to take the necessary steps to save patients’ lives. The distribution of deaths by age showed that a number of deaths were recorded in the age group of 30–39 (66%) followed by the age group 40–49 (28%). No deaths were recorded in patients who were 50 years or older, which suggests that older patients may have had a better survival rate because of their self-efficacy, or because they started the treatment early enough, but the rationale for the enhanced survival could not be established in this study.^21, 22, 24–28^

In addition, because of limitations relating to the design of this cross-sectional study, it is unclear whether the 11.1% of deaths that were undetermined were caused by other infections, or adverse effects that were not documented. Similarly, it is not known whether the non-prescribed medicines taken by some patients could have contributed to the occurrence of adverse events; this has been reported.^29^ Further prospective studies, including reported data on adverse effects, are needed to assess the impact of the involvement of other non-prescribed medicines on the deaths attributed to side effects such as lactic acidosis. Previous studies demonstrated the need for such studies.^7, 17, 29^ Professional health-care workers play an important role in the provision of antiretroviral therapy,^30^ and as such there is a need to keep them updated on new developments and to train them on the implementation of pharmacovigilance concepts in their clinical practice.

## Conclusion

In addition to tuberculosis, side effects in particular, lactic acidosis was the other main cause of death in patients treated at the study site. These findings suggest that patients who take regimens containing drugs that cause lactic acidosis should be closely monitored when the first complaints suggesting lactic acidosis, are reported or noticed.
